# Supplementary material for: Non‐apoptotic TRAIL function modulates NK cell activity during viral infection
Source: EMBO Rep. 2019 Nov 19;21(1):e48789. doi: 10.15252/embr.201948789 (PMC6945065; doi:10.15252/embr.201948789)
Supplement: Supplementary file 1 — Appendix [file EMBR-21-e48789-s001.pdf]

Appendix

Table of contents

Appendix Figure S1 2

Apoptosis-independent modulation of NK cell function by TRAIL (Graphical summary). 2

Appendix Figure S2 4

Trail-dependent expansion of virus-specific CD8$^+$ T cells is LCMV strain-specific. 4

Appendix Table S1..5

Antibodies, clones, conjugates and manufacturers 5

Appendix Figure S1

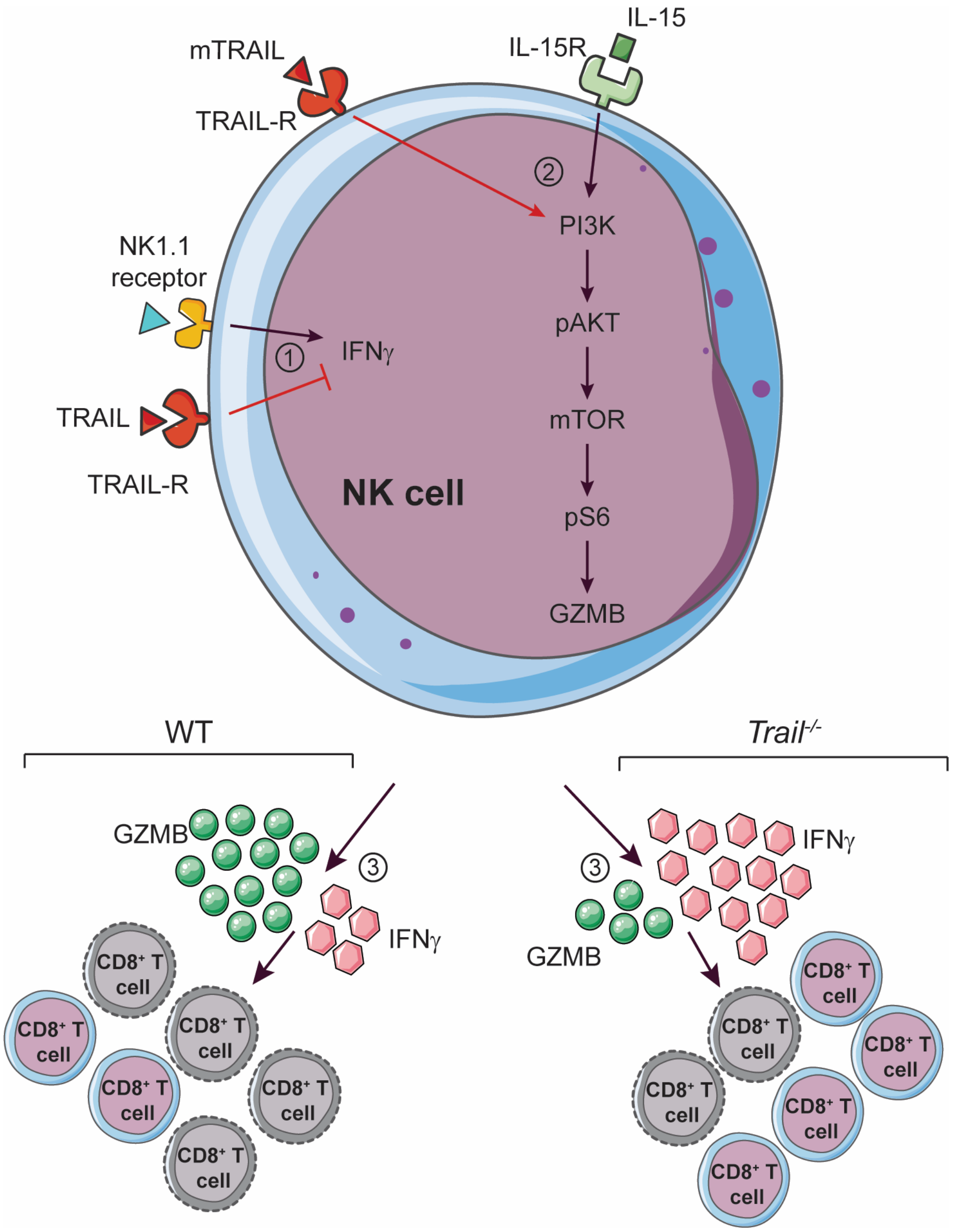

Apoptosis-independent modulation of NK cell function by TRAIL (Graphical summary).

(1) TRAIL restrains the signaling downstream of the NK1.1 activating receptor to limit IFNγ production. **(2)** TRAIL promotes the signaling downstream of IL-15/IL-15 receptor to increase granzyme B production. **(3)** The amount of granzyme B produced

by activated NK cells determines their cytotoxic potential against (LCMV-) specific $CD8^+$ T cells. It is therefore indirectly proportional to the $CD8^+$ T cell response and the virus clearance.

Appendix Figure S2

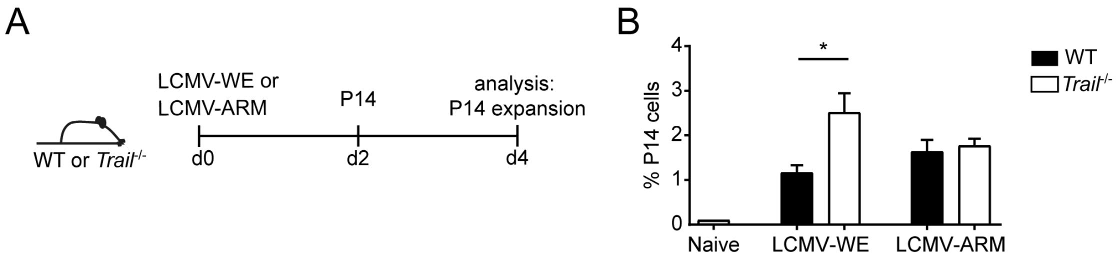

***Trail*-dependent expansion of virus-specific CD8$^+$ T cells is LCMV strain-specific.**

A. Experimental setup of P14 cell transfer experiments following infection of WT and *Trail*$^{-/-}$ mice with the indicated LCMV strains.

B. P14 cell expansion was analyzed in spleen 4 days post infection with 10^5 plaque-forming units (pfu) of LCMV (n=5-6 mice per group of infected mice). Data indicate mean ± s.e.m. Statistical analyses were performed using unpaired two-tailed *t* test. *p<0.05.

Appendix Table S1

Antibodies, clones, conjugates and manufacturers

<u>Mouse:</u>

Specificity	Clone	Conjugate	Catalog number	Source
CD107a	1D4B	FITC	121605	BioLegend
CD11b	M1/70	Pacific Blue	101224	BioLegend
CD11c	N418	PE-Cy7	117318	BioLegend
CD11c	N418	APC-Cy7	117324	BioLegend
CD122	TM-b1	APC	17-1222	eBioscience
CD3e	145-2C11	FITC	100306	BioLegend
CD3e	145-2C11	PE-Cy5	15-0031-83	eBioscience
CD4	GK15	APC	100412	BioLegend
CD4	RMA-5	PE	12-0042	eBioscience
CD4	RM4-4	Pacific Blue	116008	BioLegend
CD4	RMA-5	APC-Cy7	100525	BioLegend
CD45.1/Ly5.1	A20	PE	12-0453	eBioscience
CD45.1/Ly5.1	A20	FITC	110706	BioLegend
CD45.2/Ly5.2	104	PE-Cy7	25-0454	eBioscience
CD45.2/Ly5.2	104	Alexa Fluor 700	109822	BioLegend
CD49b	DX5	PE	108908	BioLegend
CD49b	DX5	FITC	11-5971-82	eBioscience
CD69	H1.2F3	APC	104514	BioLegend
CD80	16-10A1	PerCp Cy5.5	104722	BioLegend
CD86	Gl-1	Brilliant Violet 605	105037	BioLegend
CD8a	53-6.7	FITC	100706	BioLegend
CD8a	53-6.7	Alexa Fluor 700	100730	BioLegend
Eomes	Dan11mag	Alexa Fluor 488	53-4875-82	eBioscience
Granzyme A	GzA-3G8.5	PE-Cy7	25-5831	eBioscience
Granzyme B	GB11	Pacific Blue	515408	BioLegend
I-Ab	AF6.120.1	FITC	116406	BioLegend
IFNγ	XMG1.2	APC	505810	BioLegend
Ly49A	YE/48.10.6	FITC	116805	BioLegend
Ly49H	3D10	FITC	115886	eBioscience
Ly49I	YLI-90	FITC	115895	eBioscience
NK1.1	PK136	PE-Cy5	108715	BioLegend
NK1.1	PK136	Brilliant Violet 605	108740	BioLegend
NK1.1	PK136	APC	17-5941	eBioscience
NKG2D	CX5	APC	130211	BioLegend
NKp46	29A1.4	PE-Cy7	137617	BioLegend
NKp46	29A1.4	PerCp Cy5.5	137609	BioLegend
pAKT	SDRNR	eFluor 450	48-9715	eBioscience

pS6	D57.2.2E	PE	5316S	Cell signaling
T-bet	eBio4B10	PE-Cy7	25-5825-80	eBioscience
T-bet	eBio4B10	PerCp Cy5.5	45-5825-82	eBioscience
TNF	MP6-XT22	PE	506306	BioLegend
TRAIL	N2B2	PE	109305	BioLegend
TRAIL-R/DR5	MD5-1	PE	119905	BioLegend

Human:

**Specificity**	**Clone**	**Conjugate**	**Catalog number**	**Source**
pS6	D57.2.2E	PE	5316S	Cell Signaling
CD3	UCHT1	APC	300458	BioLegend
CD56	5.1H11	FITC	362545	BioLegend
DR4	DJR1	PE	307205	BioLegend
DR5	DJR2-4 (7-8)	APC	307407	BioLegend
Granzyme B	GB11	Pacific Blue	515408	BioLegend
TRAIL	RIK2	PE	308205	BioLegend
