# Supplementary material for: Non‐apoptotic TRAIL function modulates NK cell activity during viral infection
Source: EMBO Rep. 2019 Nov 19;21(1):e48789. doi: 10.15252/embr.201948789 (PMC6945065; doi:10.15252/embr.201948789)
Supplement: Supplementary file 2 — Expanded View Figures PDF [file EMBR-21-e48789-s002.pdf]

Expanded View Figures

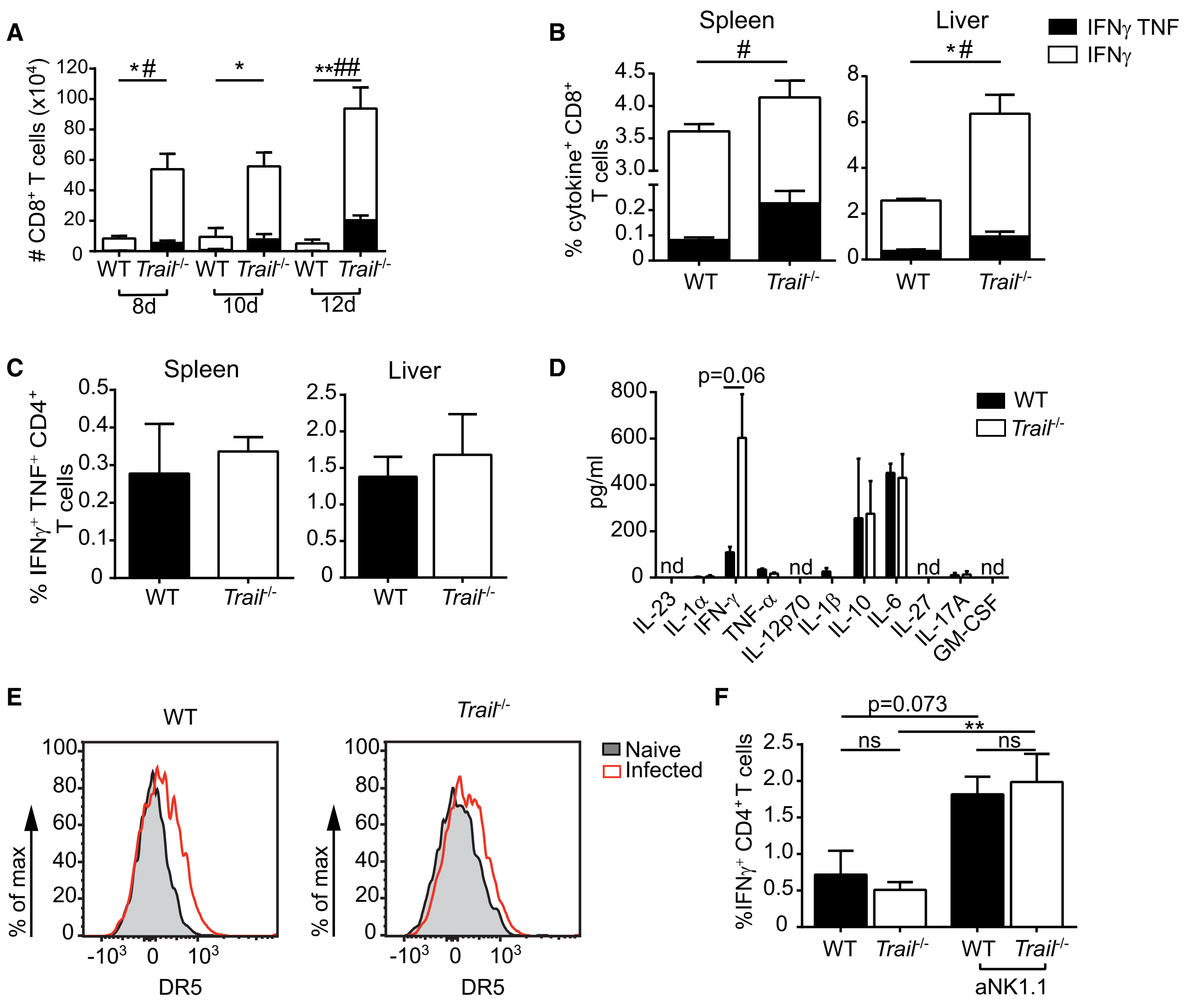

Figure EV1. ***Trail*** **deficiency leads to an altered immune response in LCMV-infected mice.**

A–C Total numbers of cytokine-producing GP_{33-41}-specific $CD8^+$ T cells were counted in the spleen at the indicated time points after LCMV infection (A). Frequencies of cytokine-producing $NP_{396-404}$-specific $CD8^+$ T cells (B) or GP_{61-80}-specific $CD4^+$ T cells (C) were measured 8 days postinfection. Data shown are mean ± SEM of $n = 3$ mice per group and are from at least 2–3 experiments. Unpaired two-tailed *t*-test was used. $^*P < 0.05$; $^{**}P < 0.01$ between $IFN\gamma^+$ cells. $^{\#}P < 0.05$; $^{\#\#}P < 0.01$ between $IFN\gamma^+$ TNF^+ cells.

D Cytokine concentrations were measured in the serum 36 h after LCMV infection using a cytokine multiplex assay. Data indicate mean ± SEM of $n = 4$ mice per group. nd, non-detectable. One experiment was performed. Statistical analyses were performed using unpaired two-tailed *t*-test.

E Mice were infected with LCMV, and DR5 was measured on splenic monocytes (defined as $CD11b^+CD11c^-Ly6C^+Ly6G^-$ cells) 24 h after infection. Naïve monocytes were used as a control, and data show one representative of $n = 3$ infected mice per group. One experiment was performed.

F Mice were infected with LCMV, and frequencies of $IFN\gamma^+$ GP_{61-80}-specific $CD4^+$ T cells were measured in the spleen 8 days postinfection. When indicated (aNK1.1), NK cells were depleted. Data shown are mean ± SEM of $n = 3$ mice per group and are representative of at least two independent experiments. One-way ANOVA with Tukey post-test was used. ns, non-significant; $^{**}P < 0.01$.

 © 2019 The Authors

Figure EV2. TRAIL signaling controls the expression of multiple genes in NK cells during early LCMV infection.

A Venn diagram showing the difference and overlap of genes that are differentially expressed in NK cells of WT versus *Trail*$^{-/-}$ mice during LCMV infection (for an adjusted *P*-value < 0.01 and absolute log2 fold change ≥ 2).

B, C Gene Ontology graph showing relationships between pathways that are associated with differentially expressed genes in NK cells of WT (B) or *Trail*$^{-/-}$ (C) mice during LCMV infection. Colored dots indicate the most semantically specific pathways, and gray dots represent connection nodes. Pathways related to inflammation are highlighted by a red ring.

Source data are available online for this figure

© 2019 The Authors

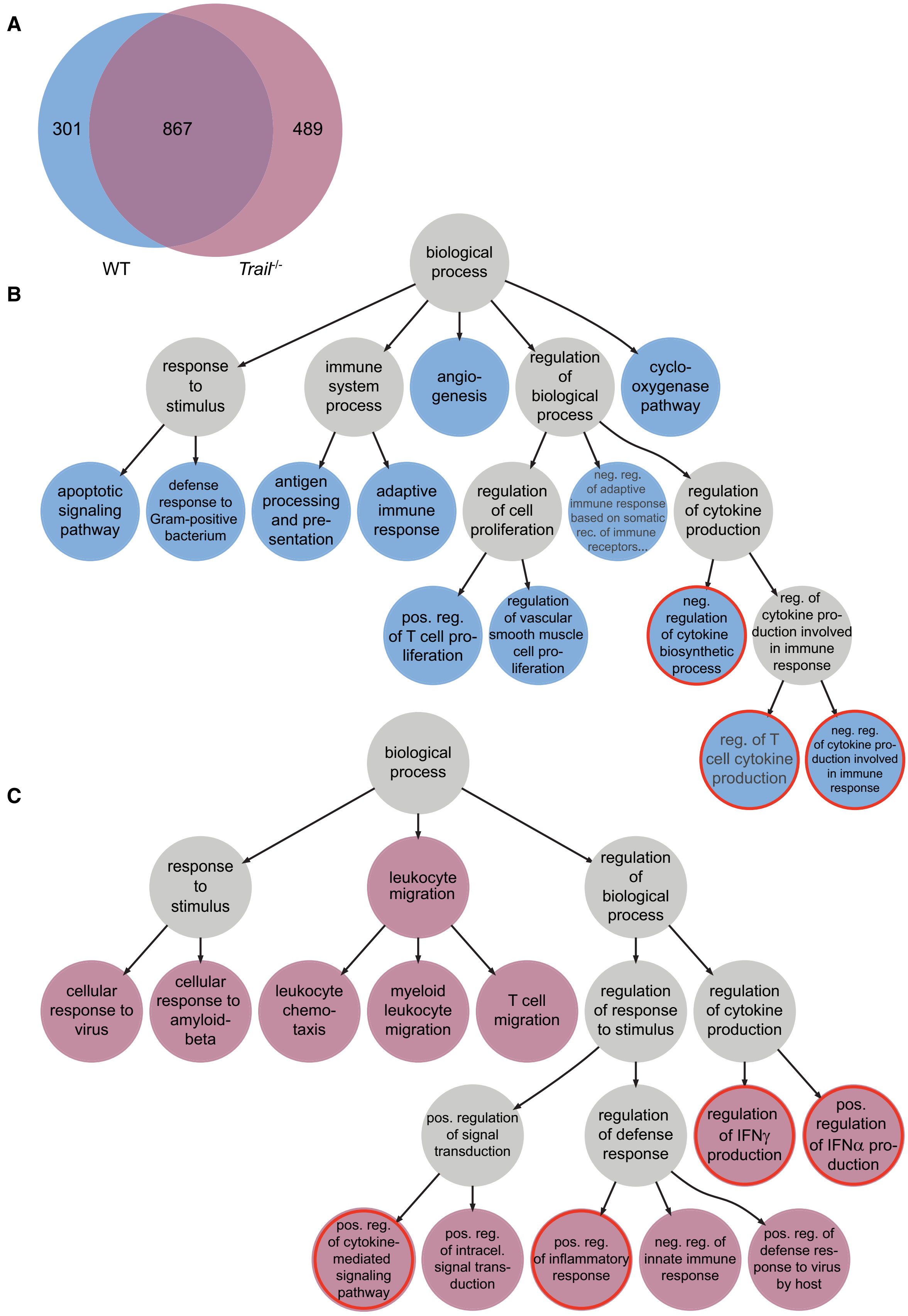

Figure EV2.

 © 2019 The Authors

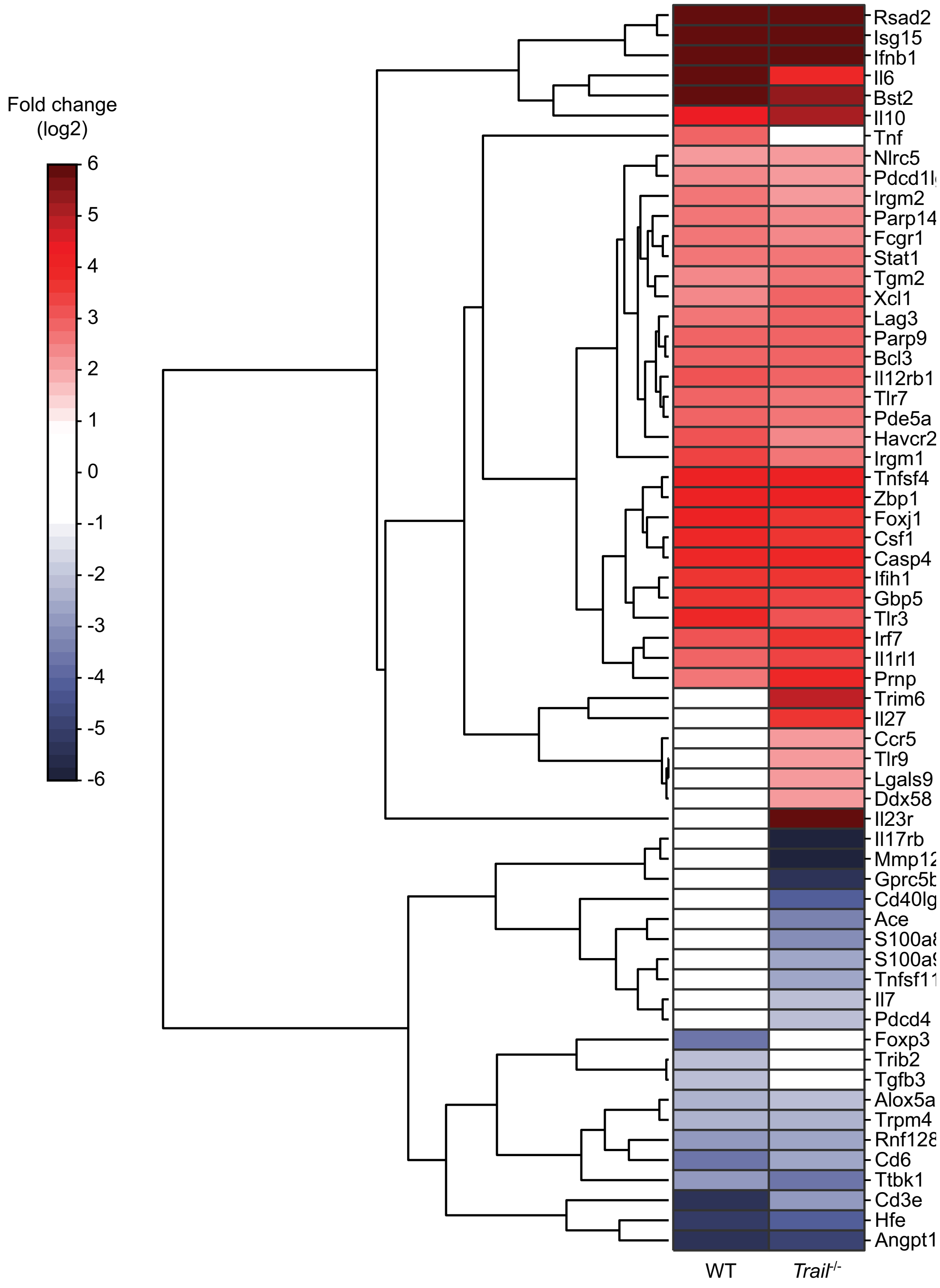

Figure EV3. TRAIL signaling controls the expression of multiple genes in NK cells during early LCMV infection.

Genes listed in the pathways highlighted by a red ring in Fig EV2 were clustered hierarchically according to their expression pattern. Each row in the dendrogram depicts the gene log2 fold changes during infection of WT or *Trail*$^{-/-}$ mice. In each sample group, dark red indicates higher and dark blue lower transcript expression.

Source data are available online for this figure

© 2019 The Authors

Figure EV4. Numbers and surface markers of NK and dendritic cells are unaltered in LCMV-infected *Trail*$^{-/-}$ mice.

A Mice were infected with LCMV and analyzed 24 h postinfection. Total numbers of NK cells were measured in the spleen and liver. Data shown are mean ± SEM of n = 3 mice per group and are representative of at least three independent experiments. Unpaired two-tailed t-test was used.

B–E Mice were infected with LCMV and analyzed 24 h postinfection. Frequencies (B) and total numbers of CD11c$^+$ dendritic cells (C) were analyzed in the spleen. Frequencies of CD11c$^+$ CD86$^+$ cells and CD86 expression on CD11c$^+$ cells (D), and frequencies of CD11c$^+$ I-A^b MHCII$^+$ cells and I-A^b MHC II expression on CD11c$^+$ cells (E) were quantified in the spleen. Data shown are mean ± SEM of n = 3 mice per group and are representative of at least two independent experiments. MFI, mean fluorescence intensity. Unpaired two-tailed t-test was used.

F–J Mice were infected with LCMV, and NK cells were analyzed 24 h postinfection. Frequencies of splenic NK cells expressing the indicated markers and mean fluorescence intensity (MFI) levels of these markers were assessed in spleen (F) and liver (G). Frequencies of Ly49H-positive NK cells were quantified by flow cytometry in the spleens of naïve mice (H). Eomes (I) or T-bet (J) expression was analyzed by measuring frequencies of positive NK cells or MFI levels 24 h postinfection. (F, G, I, J) Data shown are mean ± SEM of n = 3 mice per group from three independent experiments. (H) Data show one representative of two independent experiments, with 2 representative mice out of 3 tested per strain. Unpaired two-tailed t-test was used. *$P < 0.05$; **$P < 0.01$.

K NK cells were isolated from naïve mice, and *Gzmb* transcript levels were quantified. Data are represented as fold induction relative to *Gapdh*. Data shown are mean ± SEM of n = 3 mice per group from a unique experiment. Unpaired two-tailed t-test was used.

L NK cells from naïve mice were analyzed for CD11b and CD27 expression. DN: double-negative, CD11b^{low}CD27^{low} NK cells; CD11b^{low}: CD11b^{low}CD27^{hi} NK cells; DP: double-positive, CD11b^{hi}CD27^{hi} NK cells; CD27^{low}: CD11b^{hi}CD27^{low} NK cells. Data shown are mean ± SEM of n = 3 mice per group from three independent experiments. Unpaired two-tailed t-test was used.

 © 2019 The Authors

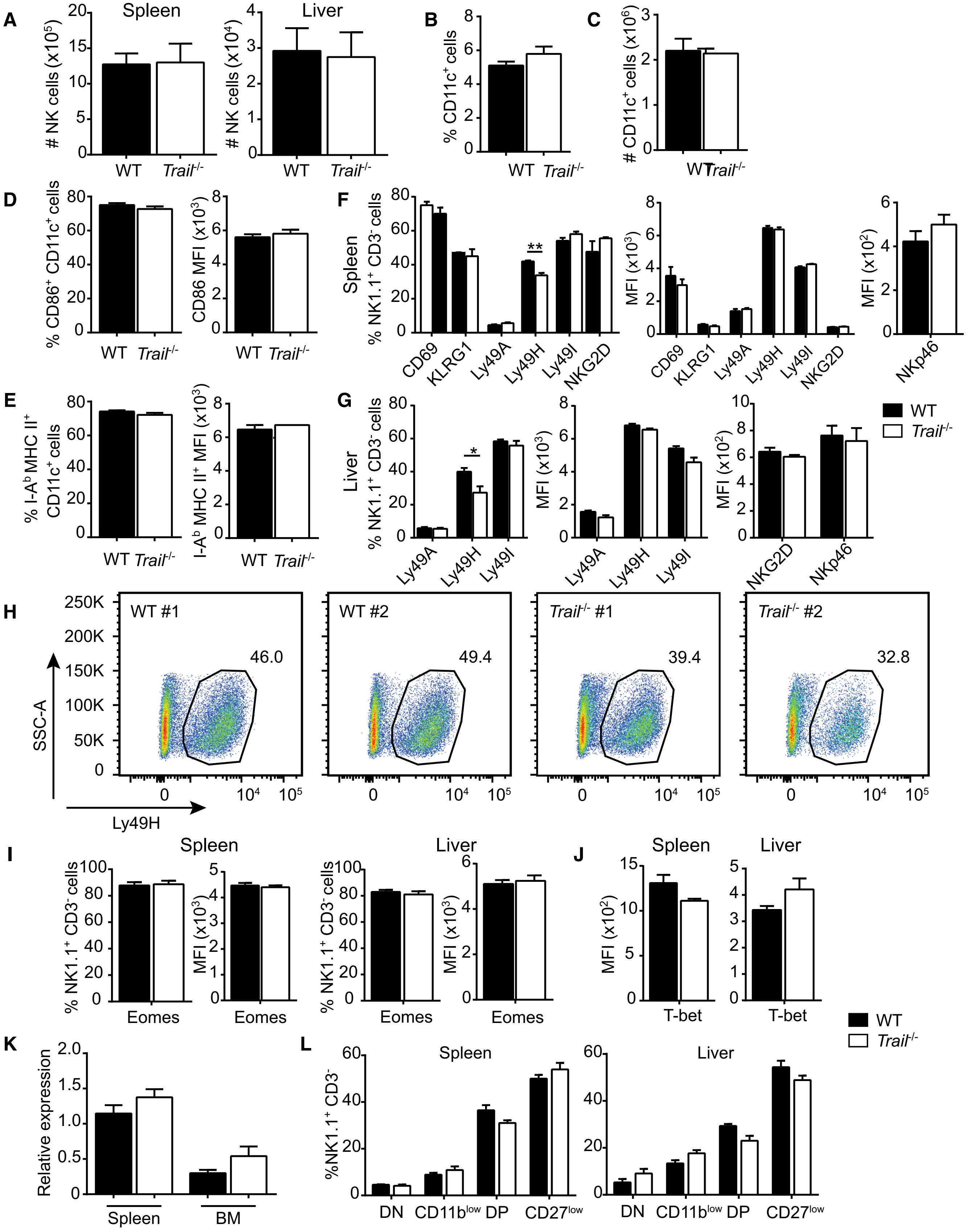

A
B
C
D
E
F
G
H
I
J
K
L
Spleen
Liver
WT
Trail-/-
WT #1
WT #2
Trail-/- #1
Trail-/- #2
46.0
49.4
39.4
32.8
SSC-A
Ly49H
Eomes
T-bet
Relative expression
BM
DN
CD11b low DP
CD27 low

Figure EV4.

Figure EV5. TRAIL regulates IL-15 and NK1.1 signaling in NK cells.

A NK cells were MACS-purified from single-splenocyte suspensions from naïve donors, stimulated *in vitro* for 1 h with IL-15, and phosphorylation of S6 was measured by flow cytometry. One representative of two independent experiments is depicted (n = 1 mouse per group).

B AKT and S6 phosphorylation were measured in naive splenic NK cells. Data shown are mean ± SEM of n = 3 mice per group from at least two independent experiments. Statistical analyses were performed using unpaired two-tailed t-test.

C Splenocytes were cultured with IL-15 ± wortmannin (WRM)/LY294002, and GZMB expression was measured in NK cells. Data shown are mean ± SEM of n = 3 mice per group from at least two independent experiments. Statistical analyses were performed using one-way ANOVA with Dunn's post-test. *$P < 0.05$; **$P < 0.01$.

D, E WT splenocytes were cultured with IL-15 ± TRAIL-R2-Fc chimeric protein, and AKT phosphorylation (D) or S6 phosphorylation (E) was measured in NK cells. Values shown were normalized to unstimulated control. Data show n = 5 mice per group, pooled from two independent experiments. Statistical analyses were performed using paired two-tailed t-test. *$P < 0.05$; **$P < 0.01$.

F–H Splenocytes from naïve WT and *Trail*$^{-/-}$ mice were cultured with the indicated cytokines, and frequencies of IFNγ^+ NK cells (F), or IFNγ-expression levels in NK cells (G) were measured after 5 h. Alternatively, naïve splenocytes were cultured with IL-18/IL-12, and IFNγ was measured in the supernatant at the indicated time points (H). Data shown are mean ± SEM of n = 3–4 mice per group and are representative of two (F, G) or three (H) independent experiments. Unpaired two-tailed t-test was used. *$P < 0.05$.

I, J MACS-purified DX5$^+$ cells were cultured in wells coated with an anti-NK1.1 antibody, and frequencies of IFNγ^+ NK cells were measured after 5 h (I) or GZMB expression was measured after 24 h (J). Data shown are mean ± SEM of n = 3–4 mice per group and are representative of three (I) or one (J) independent experiments. Unpaired two-tailed t-test was used. **$P < 0.01$.

K–O Flow cytometry was applied on NK-92 cells to assess expression of TRAIL (K) and the TRAIL receptors DR4 (L) and DR5 (M). NK-92 cells were stimulated with IL-2 ± human TRAIL-R2-Fc chimeric protein, and S6 phosphorylation (N) or GZMB expression (O) was measured by flow cytometry (n = 1 per condition). (N, O) Data show one representative of 2 independent experiments.

 © 2019 The Authors

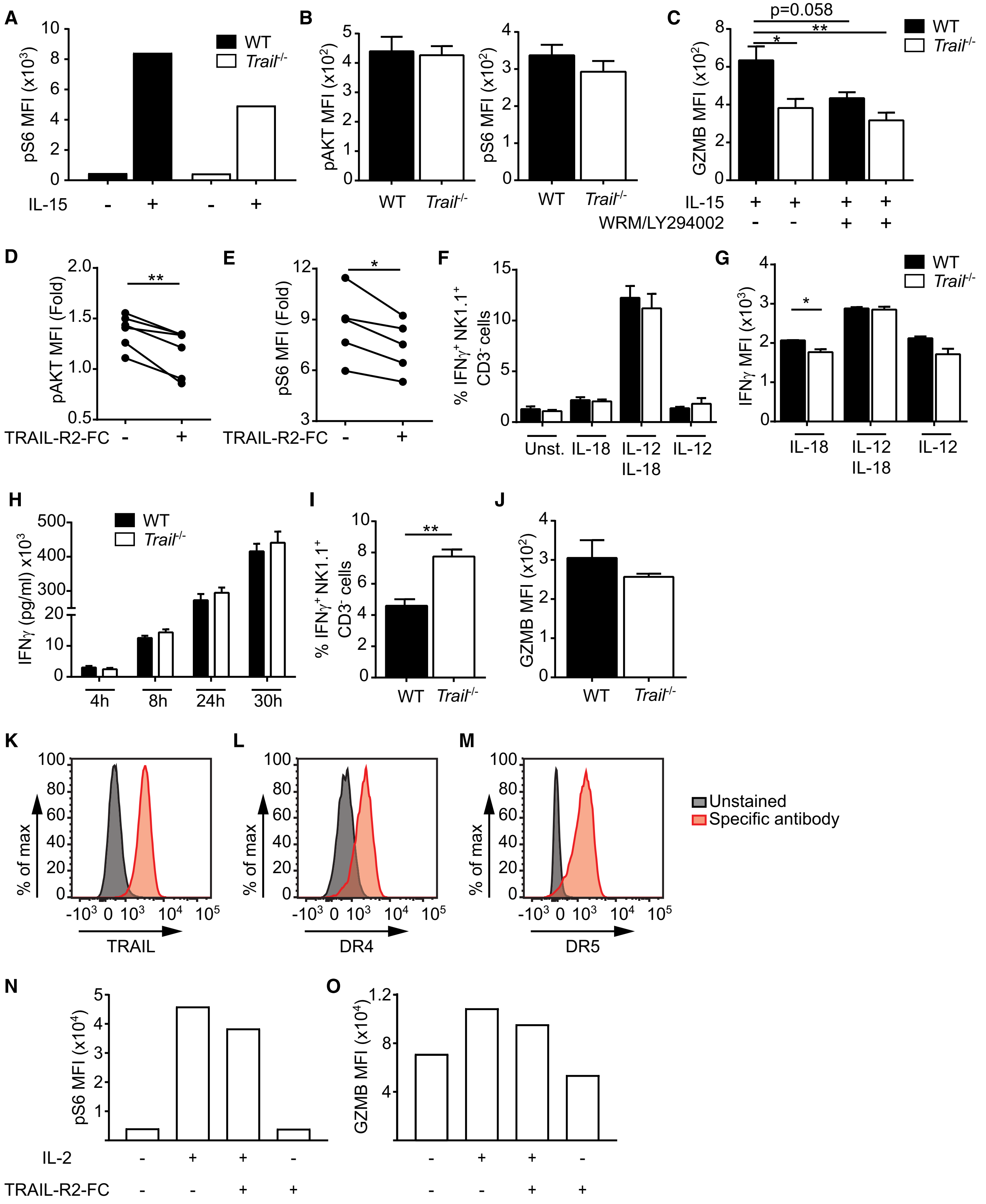
A
pS6 MFI (x10³)
WT
Trail-/-
IL-15 - + - +
B
pAKT MFI (x10²)
pS6 MFI (x10²)
WT Trail-/-
C
p=0.058
*
**
GZMB MFI (x10²)
WT
Trail-/-
IL-15 + + + +
WRM/LY294002 - - + +
D
**
pAKT MFI (Fold)
TRAIL-R2-FC - +
E
*
pS6 MFI (Fold)
TRAIL-R2-FC - +
F
% IFNγ+ NK1.1+ CD3- cells
Unst. IL-18 IL-12 IL-18 IL-12
G
WT
Trail-/-
*
IFNγ MFI (x10³)
IL-18 IL-12 IL-18 IL-12
H
WT
Trail-/-
IFNγ (pg/ml) x10³
4h 8h 24h 30h
I
**
% IFNγ+ NK1.1+ CD3- cells
WT Trail-/-
J
GZMB MFI (x10²)
WT Trail-/-
K
% of max
TRAIL
L
% of max
DR4
M
% of max
DR5
Unstained
Specific antibody
N
pS6 MFI (x10⁴)
IL-2 - + + -
TRAIL-R2-FC - - + +
O
GZMB MFI (x10⁴)
- + + -
- - + +

Figure EV5.
